# Development of Ultra-Performance Liquid Chromatography with Post-Column Fluorescent Derivatization for the Rapid Detection of Saxitoxin Analogues and Analysis of Bivalve Monitoring Samples

**DOI:** 10.3390/toxins11100573

**Published:** 2019-10-01

**Authors:** Ryuichi Watanabe, Makoto Kanamori, Hidetsugu Yoshida, Yutaka Okumura, Hajime Uchida, Ryoji Matsushima, Hiroshi Oikawa, Toshiyuki Suzuki

**Affiliations:** 1 National Research Institute of Fisheries Science, Fukuura 2-12-4, Kanazawa, Yokohama, Kanagawa 236-8648, Japan; rwatanabe@affrc.go.jp (R.W.); huchida@affrc.go.jp (H.U.); matsur@affrc.go.jp (R.M.); oikawah@affrc.go.jp (H.O.); 2 Hakodate Fisheries Research Institute, Hokkaido Research Organization, Benten-cho 20-5, Hakodate, Hokkaido 040-0051, Japan; kanamori-makoto@hro.or.jp (M.K.); yosida-hidetugu@hro.or.jp (H.Y.); 3 Tohoku National Fisheries Research Institute, Shinhama-cho 3-27-5, Shiogama, Miyagi 985-0001, Japan; okumura@affrc.go.jp

**Keywords:** saxitoxin analogues, UPLC, shellfish toxicity, UPLC/OX/FD, post-column detection, saxitoxin

## Abstract

Saxitoxin (STX) and its analogues produced by toxic dinoflagellates accumulate in bivalves, and routine monitoring of bivalves is important to prevent cases of human poisoning. In this study, we describe a rapid detection method for the analysis of STXs using ultra-performance liquid chromatography with post-column fluorescent detection and to investigate water depths and sampling points optimal for shellfish toxin monitoring. Cultured scallops (*Mizuhopecten yessoensis*) and mussels (*Mytilus galloprovincialis*) collected from various water depths and sampling points were used in this study. Irrespective of bivalve species, toxin concentrations in bivalves were lower at deeper water depths. The toxin concentrations of bivalves did not differ greatly when bivalves were collected from the same bay. Although the levels of contamination of bivalves with STXs can depend on various environmental and geographical factors, our findings are useful for formulating a sampling protocol for the prevention of harvesting contaminated shellfish.

## 1. Introduction

Saxitoxins (STXs) produced by certain toxic dinoflagellates such as *Alexandrium tamarense* [1] are accumulated through the food chain in organisms such as bivalve mollusks [2] or crabs [3,4,5,6,7]. More than 50 saxitoxin analogues are known to date [8]. Saxitoxins act on site 1 of the voltage-gated sodium channel of the nerve system and block the influx of ions into the cell [9], causing neurotoxic symptoms to mammals. Paralytic shellfish poisoning (PSP) occurs throughout the world when STX-contaminated bivalves are ingested [10,11,12].

Recently a new method (validated in a single laboratory trial [13]) for STX analysis involving the use of tandem mass spectrometry with liquid chromatography (LC/MS/MS) has been reported as a rapid and selective detection method of STXs [14]. However, the initial cost of LC-MS/MS instruments is high and is a significant factor in limiting access to the method for some laboratories, although the purchase can be justified in routine laboratories where high sample numbers are expected. Detection methods for STXs using conventional HPLC systems have been used for more than 20 years for research and as monitoring tools. These methods are classified into two groups: One is a pre-column oxidation method and the other is a post-column oxidation method. Both detection methods are registered as AOAC official methods of analysis [15,16]. The former method is based on the conversion of STXs to fluorescent derivatives prior to the chromatographic separation [17,18]. The complicated peak identification protocols to obtain each STX analogue concentrations in samples significantly limits the number of STX-positive samples that can be practically quantified by this method. The latter method [19,20] has an advantage in easy peak identifications of individual congeners and calculation of concentrations of STXs. A drawback of the latter method is that multiple injections under different chromatographic conditions are typically required to cover the various STX analogues and more rapid method in terms of chromatographic separations of STXs are required to analyze large numbers of samples.

The FAO technical report (2011) [21] describes the requirements for spatially and temporally representative sampling, and shellfish sampling size. In the report, it is written that depth-specific sampling of microalgae should be considered when a toxic event is in progress, and sampling for shellfish grown in suspension should at least involve an integrated sample composed of shellfish taken from the top, middle, and bottom of the lines [21]. However, there are no available reports on the relationship between shellfish toxicities and water depths. In addition, sampling stations should be selected based on sites that have historically experienced toxic events [21]. In the report, it is written that the selection of sampling stations should give appropriate coverage of the extent of a toxic event or the likely “worst case scenario” in a growing area [21]. However, microalgae have a “patchy” distribution [21] and it is very difficult to identify which stations are suitable in a growing area.

In this study, we aimed to develop a rapid and cost-effective ultra-performance liquid chromatograph with post-column fluorescent derivatization (UPLC/OX/FD) method for STXs. The method was then applied to clarify the relationships between shellfish toxicities and water depths, and between shellfish toxicities and geographical distribution. 

## 2. Results

### 2.1. Development of UPLC/OX/FD

#### 2.1.1. Method Development

**Figure 1:**
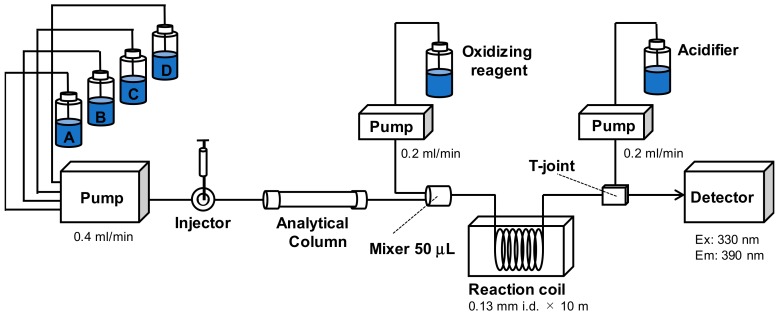
Diagram of the ultra-performance liquid chromatograph with post-column fluorescent derivatization (UPLC/OX/FD).

In the development of a rapid UPLC/OX/FD method for the detection of STXs, we improved the conventional HPLC/OX/FD method developed by Oshima, Tohoku University, Japan in 1995 [19], which has been applied worldwide for the detection of STXs in bivalves. The setup of our rapid detection method is shown in Figure 1. Our initial objective was the optimization of the fluorescent derivatization of STXs in the post-UPLC column reaction system consisting of the oxidizing reagent, acidifier, and reaction coil. We found that the fluorescent products of STXs were efficiently and reproducibly generated using a 50 μL volume mixer for the mobile phase and oxidizing reagent. Other reaction conditions were basically the same as those reported by Oshima [19]. During our optimization procedure we re-confirmed that at least a 10 m reaction coil length was required to obtain reproducible results in terms of the intensities of fluorescent products. The coil length had little effect on peak broadening.

Next, we investigated the composition of the mobile phase delivered by the quaternary pump. The mobile phase of the original method reported by Oshima [19] was developed with a single or binary pump. Three different mobile phases were used for the three toxin groups based on the ion charges in the structures. By using the quaternary pump, we aimed to develop a system that could automatically analyze all three toxin groups. To wash the column, two lines for acetonitrile and Milli-Q water were used. The C-toxins were analyzed with a different ion-pair reagent from the gonyautoxins (GTXs) and saxitoxin (STX) groups and a different single line was used for C-toxins (1 mM phosphate buffer at pH 6.3 containing 2 mM tetrabutylammonium phosphate). As GTXs and STX groups were chromatographed with the same ion-pair reagent, a stock solution for both groups was prepared as a 2 fold concentration of the GTXs’ mobile phase (10 mM phosphate buffer at pH 7.1 containing 2 mM sodium heptanesulfonate) as described in the original method. The stock solution was diluted with Milli-Q water for the elution of the GTXs group and was mixed with acetonitrile for elution of the STXs group. This mobile phase system allowed us to automatically analyze all three toxin groups including the column washing procedure. 

**Figure 2:**
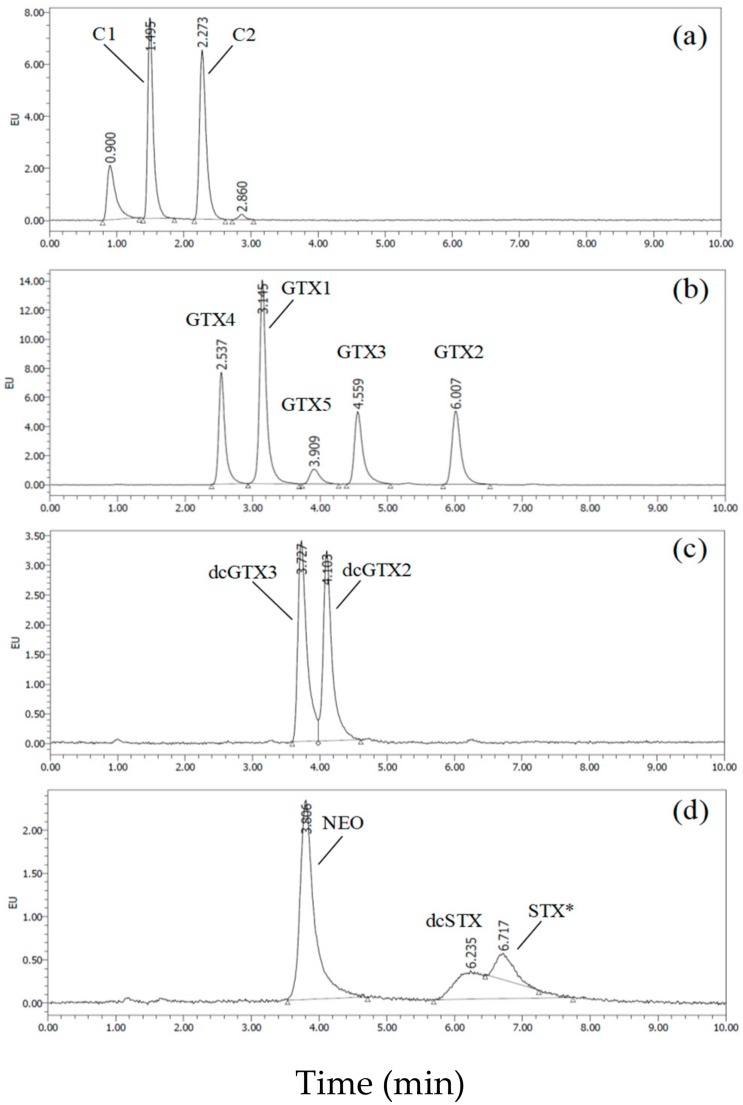
Representative chromatograms of eleven STX analogues analyzed by the UPLC/OX/FD on an HSS-C18 column (2.1 mm i.d. × 100 mm, 1.7 μm, Waters). (**a**) chromatograms of C1+2; (**b**) chromatograms of GTX1–5; (**c**) chromatograms of dcGTX2+3; (**d**) chromatograms of neoSTX and dcSTX. * The peak might be STX, although this was not confirmed because STX was not used in the study.

Representative chromatograms of eleven STXs obtained with an HSS-C18 column (Waters) are shown in Figure 2. We achieved a reduction of half the previous analytical run time of all toxin groups. In the original method, approximately 20 min were required to separate the GTXs group. In our present method, GTX1–5 were completely separated within 10 min (Figure 2). The program employed in the automatic STXs analysis using the mobile phases is shown in Table 3. When 20 samples are analyzed with our present method, the total LC run time, including equilibration and column washing, is less than 12 h. This run time is far shorter than the original Oshima method (19.5 h), although the HILIC LC/MS/MS method (4.5 h) is shorter than other methods. The reproducibility of the retention times of all the STX analogues was confirmed.

We also investigated the use of several analytical columns for the UPLC/OX/FD analysis. Multiple reverse phase columns (2.1 mm i.d. × 100 mm length) with various functional groups (C8 and C18) were suitable for all toxin groups; however, a C18-column (HSS-C18 and Cadenza CD-C18UP columns) was superior in toxin retention and separation compared to a C8-column (Unison UK-C8UP and BEH C8 columns). It was also noted that columns with insufficient endcapping (HSS-T3 column) gave broad peaks because of undesirable interactions of residual silanol functionality with N1-hydroxy toxins such as neoSTX and GTX1+4.

#### 2.1.2. Characteristics of the Rapid UPLC/OX/FD Method

This method further developed and improved on the original method reported by Oshima [19]. The method has several key advantages including: The switching among the chromatographic methods was automated, the equilibration time of the column was reduced from over 60 min to 15 min per chromatographic method, the total per sample analytical run time was reduced from 50 min to 30 min, many users that have experience with the original method would easily be able to set-up and benefit from this new method if a UPLC quaternary pump was available.

Table 1 shows the limit of detection (LOD) and limit of quantitation (LOQ) of each toxin standard in UPLC/OX/FD. The LOD ranged from 1.6 nM to 135.6 nM and the LOQ from 5.4 nM to 451.5 nM. Compared to the conventional method [19], the sensitivity was similar or superior, except for NEO and dcSTX.

### 2.2. The Toxin Concentration of the STXs in Scallop Mizuhopecten yessoensis Collected from Different Water Depths 

**Figure 3:**
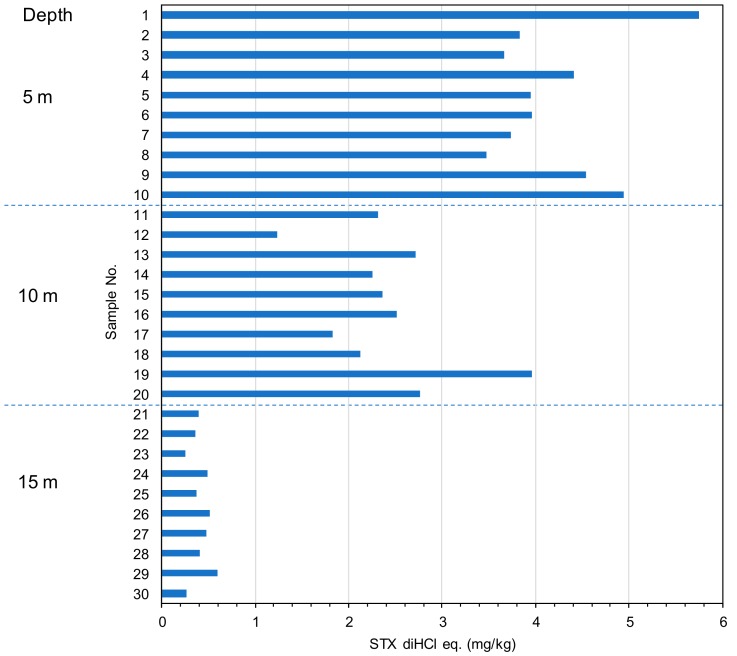
Toxin concentration of the scallop *Mizuhopecten yessoensis* collected from different water depths. Each sample number corresponds to one scallop digestive gland.

Ten individual scallops were collected from each water depth (5 m, 10 m, and 15 m) at a sampling site in Funka Bay, Hokkaido, Japan. The digestive glands taken from the whole bodies were extracted with 0.1 M hydrochloric acid according to the AOAC method. The acidic extracts were loaded on a Sep-Pak C18 plus cartridge to recover 0.5 mL of the undiluted fraction followed by the first 1.5 mL of fraction, then centrifuged to obtain the filtrate. The STXs in the filtrate were analyzed by our UPLC/OX/FD method. Figure 3 shows the toxin concentrations of the scallops collected from different water depths. The mean toxicities at 5 m, 10 m, and 15 m depths were 4.23 ± 0.7 mg/kg of STX diHCl equivalent (eq.) (16.5% rsd), 2.41 ± 0.7 mg/kg of STX diHCl eq. (29.2% rsd), and 0.41 ± 0.1 mg/kg of STX diHCl eq. (27.4% rsd), respectively. The toxin concentration of the scallops tended to be higher in the upper layer water depths. Although this tendency needs to be confirmed over the course of further monitoring, it suggests that scallops from a shallow 5 m water depth are suitable for monitoring purposes in this bay.

### 2.3. The Toxin Concentration of the STXs in Mussel Mytilus galloprovincialis Collected from Different Water Depths and Geographical Distances. 

**Figure 4:**
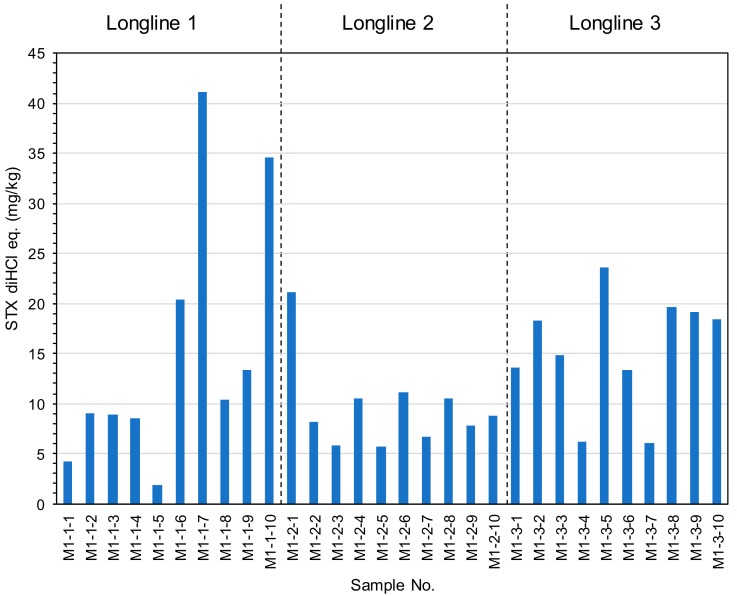
Toxin concentration in the mussel *Mytilus galloprovincialis* collected from different long-lines. Each sample number corresponds to one whole body of mussel.

**Figure 5:**
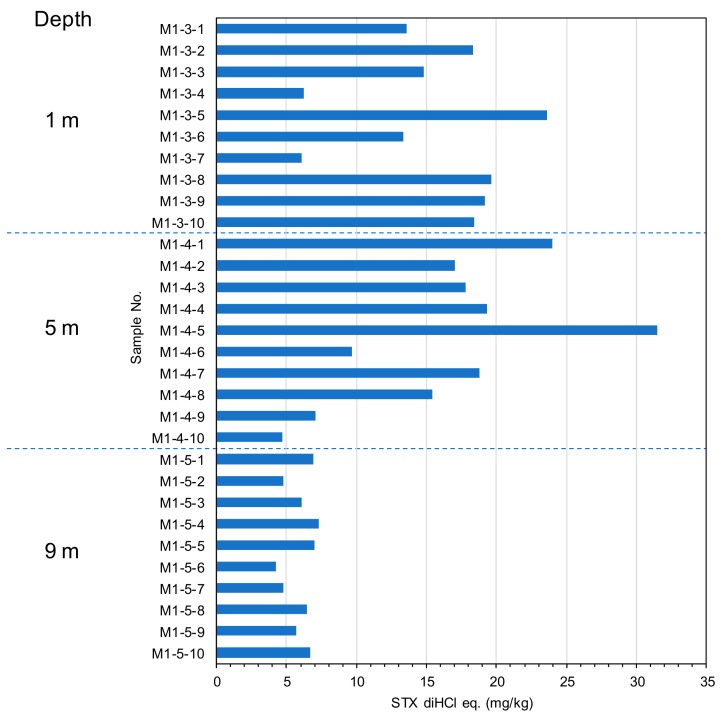
Toxin concentration in mussel *Mytilus galloprovincialis* collected from different water depths. Each sample number corresponds to one whole body of mussel.

Ten mussels from a longline were collected at 1 m depth at Ishinomaki Bay, Miyagi, Japan. The mussels were collected from three different longlines in the same bay. The distances among the three longlines were about 100 m between longline 1 and 2, and 400 m between longline 2 and 3. Whole bodies were extracted followed by the AOAC method and analyzed by UPLC/OX/FD with the same cleanup procedures as scallops. Figure 4 shows toxin concentration of the mussels collected from geographical distant longlines. The mean toxin concentrations at longline 1, 2, and 3 were 15.23 ± 13.0 mg/kg of STX diHCl eq. (85.4% rsd), 9.61 ± 4.5 mg/kg of STX diHCl eq. (49.8% rsd), and 15.31 ± 5.7 mg/kg of STX diHCl eq. (37.5% rsd), respectively, indicating that the toxin concentrations of mussels were less than 2 fold among the three different longlines. Ten individual mussels from longline 3 were collected at different water depths (1 m, 5 m, and 9 m) at Ishinomaki Bay, Miyagi, Japan. Figure 5 shows the toxin concentrations of the mussels collected from different water depths. The mean toxin concentrations at 1 m, 5 m, and 9 m were 15.31 ± 5.7 mg/kg of STX diHCl eq. (37.5% rsd), 16.53 ± 8.0 mg/kg of STX diHCl eq. (48.3% rsd), and 5.98 ± 1.1 mg/kg of STX diHCl eq. (18.1% rsd), respectively. The toxin concentrations of the mussels tended to be higher in the upper layer water depths similarly to the scallops, suggesting that mussels from 1 or 5 m shallow water depths are suitable for the monitoring purposes in this bay.

## 3. Discussion

We developed a rapid detection method for STXs using UPLC/OX/FD. As far as we know, this is the first report on a post-column reaction system using an ultra-performance liquid chromatograph. The original method reported by Oshima [19] was performed using three different analytical conditions for the three groups of congers and played an important role facilitating chemical and biochemical studies of STXs. However, the 50 min runtime per injection and the additional time required for equilibration/column washing and change of mobile phase constrains the number of sample that can be run. The runtime of our newly developed method requires 30 min. As the equilibration and column washing are automated, this can occur without additional technical attendance. The LC/MS/MS [13,14] is a more powerful tool to simultaneously determine primary STXs in toxin producers and predators, but compared to the LC/MS/MS, our method is more accessible to testing labs due to the lower cost of the instrumentation and it is not affected by MS ion source suppression and enhancement effects that are caused by matrix interferences such as salts, etc. Our method is useful for routine shellfish monitoring by conducting automatic analyses of all three toxin groups.

Toxin concentrations in bivalves collected from each water depth and longline was determined using our UPLC/OX/FD method. Table 2 shows changes in cell densities of toxic dinoflagellate *Alexandrium tamarense* at Funka Bay. The highest toxin concentrations in the scallops observed at a 5 m depth, collected on 15 June 2015, shifted with the data, showing that high cell densities of *A. tamarense* were observed at shallow water depths (~10 m). The discrepancy between the maximum cell densities of *A. tamarense* and the highest toxin concentrations of scallop could be explained by diel vertical migration of *A. tamarense*.

At Ishinomaki Bay and Funka Bay, the toxin concentrations in the bivalves were the highest at shallow water depths of 1 m and 5 m. In the case of mussels, the toxin concentrations varied greatly within the same water depths compared to scallops. The average toxin concentrations of mussel were found to be less than two-fold among the three different longlines. High variability was observed among individuals on each longline, which highlights the importance that official testing is performed on a representative sample size. In Japan official testing requires 200 g of shellfish meat, which would average the variability of the individual toxicities.

Water depths in the sampling procedure of cultured shellfish for shellfish toxin monitoring are not well defined. Probably, shellfish toxicity has been determined so far by integrating shellfish from various water depths; however, it is desired that toxicities of shellfish should be monitored at the highest toxicity point in water depths. 

## 4. Conclusions

We developed a rapid detection method for STXs using UPLC/OX/FD. As far as we know, this is the first report on a post-column reaction system using an ultra-performance liquid chromatograph. Differences in toxin concentrations of bivalves collected from each water depth at Funka Bay and Ishinomaki Bay, Japan were confirmed using the UPLC/OX/FD method we developed. Our observations of variations in toxin concentration in shellfish at different depths and locations are the first data of this type in this monitoring area in Japan. Although further data still needs to be acquired, this finding may lead to new regulations regarding the sampling procedures for shellfish toxin monitoring in Japan. 

## 5. Materials and Methods 

### 5.1. Chemicals and Apparatus

An ultra-performance liquid chromatograph H-class including quaternary solvent manager (QSM), sample manager-FTN (SM-FTN), column oven, and FLR detector (Waters, Milford, MA) was used for detection of STXs in bivalves. The temperature control module II (Waters, Milford, MA) was set to 85 °C for generating fluorescent products of STXs. The reaction coil was composed of a PEEK tube with 0.13 mm i.d. × 10 m. A double plunger pump (SPD-2502U, Nihon Seimitsu Kagaku, Saitama, Japan) was used for pumping the oxidizing reagent and acidifier. A solvent mixer with 50 μL volume (Cat. No. 700002911, Waters, Milford, MA, USA) was used for mixing the mobile phase and oxidizing reagent.

Standards (C1+2, GTX1–5, dcGTX2+3, neoSTX, and dcSTX) of STXs prepared by Oshima, Tohoku University, were used for quantitation and identification of their toxins [19]. The toxin concentration of standards used in the study were 1.23 (C1), 0.33 (C2), 1.52 (GTX1), 0.44 (GTX2), 0.15 (GTX3), 0.52 (GTX4), 0.70 (GTX5), 0.32 (dcGTX2), 0.09 (dcGTX3), 2.98 (NEO), and 0.65 μM (dcSTX), respectively. Milli-Q water with 18.2 MΩ·cm and TOC: 3 ppb made by a Milli-Q reference device (Merck, Darmstadt, Germany) was used. Sodium heptane sulfonate (Tokyo Chemical Industry, Tokyo, Japan) and tetrabutylammonium phosphate (Sigma–Aldrich Japan, Tokyo, Japan) were used as ion-pair reagents. Ortho-periodic acid (Fujifilm-Wako, Osaka, Japan) was used for generating fluorescent products of the toxins. The analytical columns used were: HSS-C18 column (2.1 mm i.d. × 100 mm length, 1.7 μm particle size, P/N:186002352, Waters, Milford, MA, USA), HSS-T3 column (2.1 mm i.d. × 100 mm length, 1.8 μm particle size, P/N:186003539, Waters, Milford, MA, USA), BEH C8 column (2.1 mm i.d. × 100 mm length, 1.7 μm particle size, P/N:186002878, Waters, Milford, MA, USA), Unison UK-C8UP column (2.0 mm i.d. × 100 mm length, 3 μm particle size, P/N:CD024U, Imtakt, Kyoto, Japan) and Cadenza CD-C18UP column (2.0 mm i.d. × 100 mm length, 3 μm particle size, P/N: UK824U, Imtakt, Kyoto, Japan).

### 5.2. Specimens and Sample Preparation

Ten individual scallops (*Mizuhopecten yessoensis*) from Hokkaido (location: N42°16′33”, E140°20′00”), Japan were collected at three water depths (5 m, 10 m, and 15 m) in order to investigate toxicity differences of bivalves in water depths. The sampling date was 15th June 2015.

Ten individuals of mussels *Mytilus galloprovincialis* from Ishinomaki Bay, Miyagi, Japan were collected at 1 m water depth from three different longlines in order to investigate spatial differences in toxicity. Each longline was located as follows: Longline 1: N38°22′29”75–86, E141°26′38”17–27; longline 2: N38°22′28”24–28, E141°26′33”71–88; longline 3: N38°22′19”12–14, E141°26′18”01–07. In longline 3. Ten individual mussels were also collected from different water depths (1 m, 5 m, and 9 m) in order to investigate toxicity differences in water depths.

The digestive glands dissected from each scallop and mussel whole tissues were extracted using 0.1 M HCl according to the official testing method of AOAC [23]. The acidic extract obtained of 2.5 mL was applied to Sep-pak C18 plus cartridge (Waters, Milford, MA) preconditioned with 10 mL of methanol and Milli-Q water. The first 1.5 mL volume was discarded, and the next 0.5 mL was collected to the centrifugal filter unit (10 kDa cut-off, Japan-Millipore, Tokyo, Japan) [19]. The filtrate obtained by centrifugation was injected into the UPLC/OX/FD.

### 5.3. Toxin Detection

The toxins in bivalve extracts were detected using a Waters UPLC H-class coupled with post-column fluorescent derivatization, and HSS-C18 column with 2.1 mm × 100 mm at room temperature (approximately 25 °C). The mobile phases A to D used were as follows—A: 20 mM phosphate buffer at pH 7.1 containing 4 mM sodium heptane sulfonate, B: MilliQ water, C: 1 mM phosphate buffer at pH 6.3 containing 2 mM tetrabutyl ammonium phosphate, and D: Acetonitrile. Those mobile phases were mixed according to the toxins to be detected and were delivered at a flow rate of 0.4 mL/min. The mobile phase for the GTX group consisted of lines A/B (50%/50%), for the STX group lines A/D (93%/7%), and for the C-toxin group line C (100%). The program for the automated PST analyses using four mobile phases is shown in Table 3. The post column oxidizing reagent and acidifier were 50 mM potassium phosphate buffer at pH 9.0 containing 7 mM periodic acid and 0.5 M acetic acid, respectively. Those solvents were delivered at a flow rate of 0.2 mL/min. The injection volume was 3 μL. The analytical run time was 10 min for all toxin groups. The toxins were monitored at an excitation wavelength of 330 nm and an emission of 390 nm. The sample toxin concentration was calculated using toxicity equivalent factors determined by Oshima [19] and toxin concentrations obtained by UPLC/OX/FD.

## Figures and Tables

**Table 1:** Limit of detection (LOD) and limit of quantitation (LOQ) of each toxin standard in the UPLC/OX/FD analysis.

Toxins	LOD ^1^ (nM)	LOQ ^2^ (nM)		Toxins	LOD ^1^ (nM)	LOQ ^2^ (nM)
C1	10.6	35.2		dcGTX3	1.6	5.4
C2	3.3	11.1		dcGTX2	6.1	20.5
GTX4	4.2	13.9		NEO	84.0	279.9
GTX1	6.9	23.1		dcSTX	135.6	451.9
GTX5	43.4	144.7				
GTX3	1.8	6.0				
GTX2	5.3	17.5				

^1^*s*/*n* = 3 at 3 μL injection, ^2^ *s*/*n* = 10 at 3 μL injection.

**Table 2:** Changes in cell density (cells/L) of toxic dinoflagellate *Alexandrium tamarense* at Funka Bay, Hokkaido, Japan in the year 2015 ^1^.

Water Qualities		8 April	18 May	15 June	21 July	1 September
Water temperature (°C) ^2^		3.9	6.6	9.8	12.9	19.9
Salinity (psu) ^2^		31.77	31.99	31.88	32.04	32.36
Clearance (m)		4.0	4.0	6.0	13.0	8.0
Depth (m)	0	0	340	10	0	0
	5	0	360	40	0	0
	10	0	600	640	0	0
	15	0	70	80	0	0
	20	0	0	0	0	0
	25	0	0	10	0	0
	30	0	10	0	0	0

^1^ The table was prepared based on plankton information disclosed in the website of the Hakodate Fisheries Research Institute [22]. ^2^ Water temperature and salinity at each depth were also obtained. The values at each depth were averaged.

**Table 3:** Program of automatic PST analyses using four mobile phases.

Steps	Ratio of Mobile Phases (%)				Time (min)
	A	B	C	D	
Equilibrate	50	50	0	0	15
GTXs analyses	50	50	0	0	10 × *n*
Equilibrate	93	0	0	7	15
STXs analyses	93	0	0	7	10 × *n*
Column wash	0	100	0	0	10
	0	50	0	50	15
	0	100	0	0	10
Equilibrate	0	0	100	0	10
Cs analyses	0	0	100	0	10 × *n*

A: 20 mM phosphate buffer at pH 7.1 containing 4 mM sodium heptane sulfonate; B: Milli-Q water; C: 1 mM phosphate buffer at pH 6.3 containing 2 mM tetrabutyl ammonium phosphate; D: Acetonitrile; *n*: The number of analytical samples.
